# Invasive Mutualists Erode Native Pollination Webs

**DOI:** 10.1371/journal.pbio.0060031

**Published:** 2008-02-12

**Authors:** Marcelo A Aizen, Carolina L Morales, Juan M Morales

**Affiliations:** Laboratorio Ecotono, Centro Regional Universitario Bariloche, Universidad Nacional del Comahue, Bariloche, Río Negro, Argentina; University of Tennessee, United States of America

## Abstract

Plant–animal mutualisms are characterized by weak or asymmetric mutual dependences between interacting species, a feature that could increase community stability. If invasive species integrate into mutualistic webs, they may alter web structure, with consequences for species persistence. However, the effect of alien mutualists on the architecture of plant–pollinator webs remains largely unexplored. We analyzed the extent of mutual dependency between interacting species, as a measure of mutualism strength, and the connectivity of 10 paired plant–pollinator webs, eight from forests of the southern Andes and two from oceanic islands, with different incidences of alien species. Highly invaded webs exhibited weaker mutualism than less-invaded webs. This potential increase in network stability was the result of a disproportionate increase in the importance and participation of alien species in the most asymmetric interactions. The integration of alien mutualists did not alter overall network connectivity, but links were transferred from generalist native species to super-generalist alien species during invasion. Therefore, connectivity among native species declined in highly invaded webs. These modifications in the structure of pollination webs, due to dominance of alien mutualists, can leave many native species subject to novel ecological and evolutionary dynamics.

## Author Summary

Plant–animal mutualisms are characterized by weak or asymmetric mutual dependences between interacting species, such that if a plant species depends strongly on an animal species, the animal typically depends weakly on the plant, and vice versa. This limited reciprocal dependency, or “mutualism strength,” might increase species persistence by buffering plant and animal species against the extinction of any of their partners. Many plant–pollinator networks include a fraction of alien species, and it is not clear how these invaders might affect the structure of pollination webs. We analyzed 10 paired plant–pollinator webs, eight from forests of the southern Andes and two from oceanic islands, with different incidences of alien species. Highly invaded webs exhibited, on average, weaker mutualistic interactions, and hence a potential increase in network stability, than less-invaded webs. This was due to a disproportionate increase in the participation of some alien species in the most asymmetric interactions and their role as central nodes in the structure of the most invaded pollination webs. The increase in alien dominance involves the usurpation of interaction links, decreasing connectivity among native mutualists. Thus, many native species that rely on native generalists for either reproduction or survivorship become highly dependent on these super-generalist alien mutualists.

## Abstract 3

Alien mutualists can alter the structure of native pollination networks by usurping interaction links, thereby becoming central nodes of highly invaded webs.

## Introduction

Plant–animal mutualisms are highly asymmetric, such that if a plant species depends strongly on an animal species, the animal typically depends weakly on the plant, and vice versa [1,2]. Thus, the resulting mutualistic webs have a nested structure—a robust property of this type of networks [3] —whereby specialists interact preferentially with generalists, rather than with other specialists, and interactions between generalist partners form the network core [2,4]. This limited reciprocal dependence or mutualism strength might increase web stability, buffering plant and animal species against the extinction of any of their partners [1,5–8]. Additionally, a decrease in mutualism strength may indicate changes in network architecture whereby some components of an interaction network become more weakly connected, or disconnected, whereas others become more central. Here we show that integration of invasive mutualists into native plant–pollinator networks, while it does not alter overall web connectivity, decreases mutualism strength by increasing the concentration of interaction links in a few alien species.

Given the arrival of propagules of alien organisms in a new locality, invasion is usually triggered by different types of mostly human-related disturbances and/or promoted by an enemy-free space that creates appropriate conditions for establishment [9,10]. Once established, aliens can increase in abundance and even dominate an entire community through a series of direct and indirect facilitative and self-perpetuating mechanisms [9–11], which can cause displacement of native competitors and disruption of their interactions [12–15]. In particular, the fate of alien flowering plants and flower-visiting animals in a novel environment may depend largely on how well they integrate into existing pollination webs [16]. If they integrate poorly—due, for instance, to a lack of coevolutionary history with their native counterparts—then their success, in terms of seed production for plants and nectar and pollen acquisition for pollinators, may be conditioned by the presence of other alien partners. However, absolute failure of aliens to integrate into native pollination webs seems unlikely, because many plant–pollinator interactions are rather unspecific and diversified, and alien mutualists have a high chance of interacting with native generalists [17,18]. In any event, preferential interaction between alien partners might create a separate network compartment with little effect on the structure of the original native web. On the other hand, because many invasive plants and pollinators are themselves highly generalist, their interactions with other alien and native species could become central in the structure of modified plant–pollinator webs.

Alien integration need not to alter the architecture of former pollination networks in terms of its connectivity, or who interacts with whom. For instance, alien plants may compete with native plants for space and resources (light and water) but not necessarily for pollinators, whereas alien and native flower-visiting animals may present different activity periods or exploit different floral resources [19–21]. However, as invasion progresses, some alien mutualists may become increasingly abundant and/or change their per capita interaction strength, elevating their chance of interacting with a large number of partners [10,22,23] and, as long as network connectivity remains constant, of “sequestering” interaction frequency and links from the original web. The transfer of interactions from native to alien generalists might create a positive feedback that fosters invasiveness and subjects native species to novel ecological and evolutionary dynamics [11].

We explored the effects of invasive species on the structure of pollination networks by compiling information provided by 10 paired, quantitative, plant–pollinator webs, eight from the temperate forests of the southern Andes and two from oceanic islands, which differ in the functional incidence of alien species. First, we evaluated the degree of mutual dependence between interacting partners (i.e., mutualism strength), characterizing networks with different levels of invasion. Second, we assessed whether a decrease in mutualism strength found in the most invaded networks was accompanied by a shift in the identity, from native to alien, of the generalist partners participating in the most asymmetric interactions and also by differences in the ecological role played by native and alien mutualists. Finally, we investigated whether an increase in alien dominance could result in a loss of interactions and decrease in connectivity between native partners. We report that although alien species could behave as mostly unnoticed commensals during initial stages of invasion, during later stages they monopolize interactions, including those that previously formed the core of native pollination webs.

## Results

### The Pollination Webs

The 10 pollination webs included in this study varied in the total number of interacting species (i.e., their sizes) and number of alien species recorded (Table 1). The sizes of the study webs (21–69 species) and number of interactions (23–145 links) were smaller than those of many tropical pollination webs [24] or webs assembled from observations collected over large areas or over long time periods [18], but typical of local webs from other temperate regions and isolated islands [24–26]. In addition, these are the few webs studied, so far, that include both alien mutualists and some estimate of interaction frequency, a measure that relates strongly to plant reproductive success and is presumably associated with the amount of floral resources gathered by a given pollinator species [27].

**Table 1:**
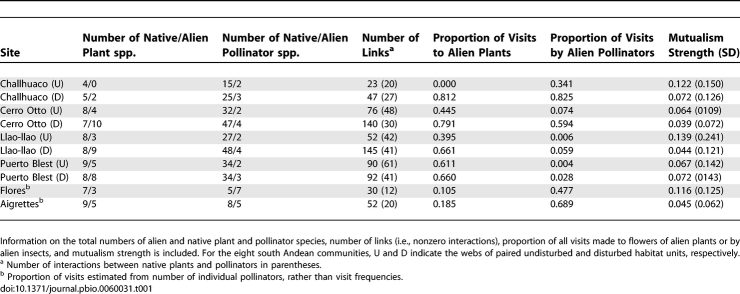
Characteristics of the Ten Plant–Pollinators Webs Analyzed

The functional importance of invading plants and animals in each pollination web was estimated from the proportions of the sum of all visitation frequencies recorded at the flowers of alien plants and for alien flower visitors, respectively (Table 1). These estimates are determined by both the number of alien species present in each web and the total interaction frequency of each individual alien species. We should acknowledge, however, that the per visit efficiency in pollen delivery or in resource uptake could differ between native and alien species and might accentuate any expected effect of invasive species on either plant or animal fitness based on changes in interaction frequency alone [15]. For all five pairs of webs, the four pairs from the southern Andes and the pair from oceanic islands, the web with the highest incidence of visits to alien plants also had the highest incidence of visits from alien pollinators (Table 1). We considered the average between these two proportions as an index of the degree of invasion of a pollination web, which ranges from 0 for a web with no interacting alien species to 1 for a web characterized exclusively by interactions between alien species. Despite extensive variation, the four southern Andean webs from mostly undisturbed habitats and the web from Flores island represent the five lowest values of this index (<0.32). These five webs were grouped into the “lightly-invaded” category, whereas their paired counterparts, including the web from Aigrettes, were grouped into the “highly-invaded” category (invasion index > 0.32).

### Invasion and Mutualism Strength

We calculated the mutual dependency for all pairs of interacting species in each web based on estimates of interaction frequency, and we consider the mean of all pairwise nonzero products of mutual dependencies as a measure of mutualism strength [1,28]. Each estimate of mutualism strength was compared with a distribution of expected values generated by a randomization procedure, and observed values were standardized by their respective expected means to lessen the influence of web size and total number of links (Table 2).

**Table 2:**
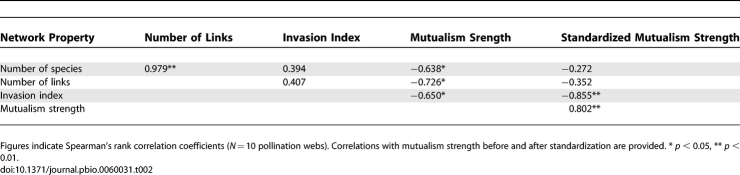
Correlations between Total Species Number, Number of Links, Invasion Index, and Mutualism Strength

**Figure 1:**
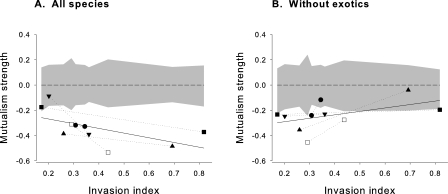
Relation of Mutualism Strength to Invasion Index for Ten Pollination Networks Mutualism strength was estimated by averaging the product of the mutual dependences over (A) all pairs of interacting species sampled in each web and (B) only pairs of interacting native species. The invasion index was the average proportion of the total interaction frequency represented by alien plants and alien pollinators. For each web, mutualism strength was standardized by the mean of the distribution of values generated by random re-distribution of observed interaction frequencies (see Materials and Methods). In each panel, symbols depict observations for the five pairs of webs analyzed (, Challhuaco; ▴, Cerro Otto; ▾, Llao-llao; •, Puerto Blest; □, Oceanic islands). Solid lines indicate the best linear fits (*y* = −0.18 – 0.42*x*, *r* ^2^= 0.427, *F* = 5.95, *df* = 1, 8, *p* = 0.041 for (A), and *y* = −0.34 + 0.27*x*, *r* ^2^= 0.250, *F* = 2.67, *df* = 1, 8, *p* = 0.14 for (B)). *N* = 10 pollination webs. Dotted line segments join points representing webs from paired disturbed and undisturbed areas in the south-Andean forest region or the two webs from oceanic islands. The gray zone is the region delimited by the 2.5 and 97.5 percentiles from the standardized random distributions.

The studied pollination webs exhibited generally smaller mean products of mutual dependences between interacting species than expected by chance alone. All but two webs, both from the lightly-invaded group, had standardized mutualism strengths below the limit set by the 2.5 percentile of their respective randomly generated distributions (Figure 1A). The highly-invaded web of each of the five network pairs had consistently lower standardized mutualism strength than its lightly invaded counterpart (binomial test, *p* = 0.03125). More generally, mutualism strength—either standardized or not—varied inversely with the extent of invasion (Table 2 and Figure 1A). This declining trend can be attributed directly to the effects of interacting alien species, as the mutualism strength of the sub-web formed by the native species showed a weak positive association with invasion index (Figure 1B). As a consequence, the (standardized) mutualism strength of the native sub-web was relatively similar to the mutualism strength exhibited by the whole network for the lightly invaded webs (mean ± standard error [SE] = −0.31 ± 0.043 versus −0.25 ± 0.047; paired *t*-test, *t* = −1.40, degrees of freedom [*df*] = 4, *p* = 0.23), whereas it was much larger for the highly invaded group (mean ± SE = −0.17 ± 0.042 versus −0.43 ± 0.047; *t* = −5.05, *df* = 4, *p* <0.01). Hence, the presence of invasive alien mutualists in highly invaded pollination webs apparently decreased absolute and relative mutualism strength.

### Invasion and the Distribution of Asymmetries

To identify the causes of changes in mutualism strength with increasing invasion, we compared the mean and distribution of the asymmetry between interacting pairs of plant–pollinator native species versus interacting pairs that included at least one alien partner for lightly and highly invaded webs. We used Bascompte et al.'s index of asymmetry, which ranges between 0 and 1 [1].

**Figure 2:**
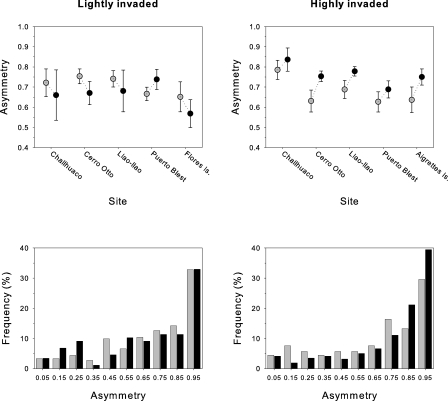
Means and Composite Frequency Distributions of Interaction Asymmetry for Pairs of Interacting Species The graphs depict asymmetries for interactions recorded in lightly invaded and highly invaded webs. Gray dots (upper panels) and bars (lower panels) indicate asymmetries for pairs of interacting native species, and black dots and bars depict pairs of interacting species in which at least one is alien. Sample sizes for each mean and frequency distribution can be derived from the column “Number of links” in Table 1. Error bars represent one standard error.

Differential asymmetries characterized the interactions involving aliens according to the extent of invasion. Whereas we did not find a significant difference between the mean asymmetry of native–native interactions and interactions involving at least one alien species for the lightly invaded webs (paired *t*-test, *t* = 1.48, *df* = 4, *p* = 0.21), we did find a consistent trend for the highly-invaded webs (*t* = −6.31, *df* = 4, *p* <0.005). For these latter webs, interactions involving alien species were more asymmetric than interactions between native mutualists (Figure 2).

The comparative distribution of pairwise interaction asymmetries also illustrates the differential influence of alien species in the structure of pollination webs. Figure 2 shows an overrepresentation of large asymmetries, implying that a strongly dependent mutualist (i.e., a specialist) commonly interacts with a weakly dependent partner (i.e., a generalist). However, the distribution of asymmetries of interactions between native mutualists and interactions involving at least one alien partner showed a differential pattern according to invasion level. Whereas the distribution of asymmetries between these two types of interactions was similar for the lightly invaded webs (Kolmogorov-Smirnov test, *D* = 0.113, *p* = 0.42), it greatly differed for the highly-invaded group (*D* = 0.184, *p* < 0.005). Specifically, in this latter group, the incidence of high asymmetry (>0.8) associated with interacting aliens was larger than those characterizing interactions between native species (60.6% versus 42.7%; χ^2^ = 13.53, *df* = 1, *p* < 0.0005), a trend that was not observed among the lightly-invaded webs (44.3% versus 47.0%; χ^2^ = 0.17, *df* = 1, *p* = 0.67). Therefore, aliens engage disproportionately in the most asymmetric interactions during advanced stages of invasion.

The differential participation of aliens in highly asymmetric interactions relates to the high individual strength that generalist alien species achieved in the most-invaded webs. We characterized each species present in each web by the number of species with which it interacts (degree) and the sum of the dependences of the species with which it interacts (strength). Thus, a species' strength is a quantitative extension of its degree, and it represents the ecological importance of a given mutualistic plant or animal species from the perspective of the interacting animal or plant assemblage, respectively [1]. We examined whether the slope of the linear relation between species degree and strength differed between native versus alien species in lightly and highly invaded webs. Alien species will exhibit a higher slope than native species if they become disproportionately important, in terms of their strength, particularly in highly invaded webs.

**Figure 3:**
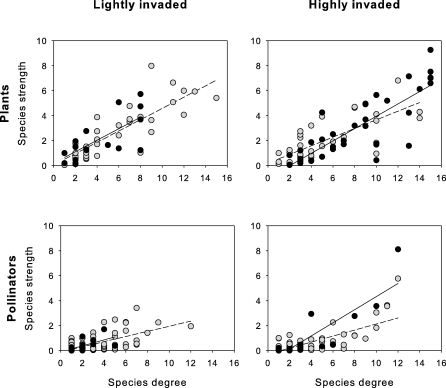
Relation of a Species' Strength to Its Degree A species' strength is the sum of the dependences of the species with which it interacts, whereas its degree is the number of species with which it interacts. The figure depicts separate relations for native (gray circles) and alien (black circles) plant and pollinators species present in lightly and highly invaded webs. The dashed and continuous lines represent the best-fit linear regressions for native and alien species, respectively (*p* < 0.001 in all cases). Sample sizes for natives and aliens are, respectively, 36 and 15 for plants in lightly invaded webs, 37 and 34 for plants in highly invaded webs, 113 and 15 for pollinators in lightly invaded webs, and 162 and 19 for pollinators in highly invaded webs.

The analysis of the relation between species strength and degree for native versus alien plant and flower visitors reveals the differential ecological importance achieved by alien mutualists along invasion (Figure 3). In the lightly invaded webs, native and alien plant and animal mutualists had almost identical slopes (test of homogeneity of slopes, *F* = 0.001, *df* = 1, 47, *p* = 0.94, and *F* = 0.13, *df* = 1, 124, *p* = 0.72 for plant and animal species, respectively). However, in the highly invaded webs, where alien species engaged in the most generalized interactions (species degree > 8), aliens exhibited a higher slope than natives (*F* = 3.45, *df* = 1, 67, *p* = 0.06, and *F* = 42.44, *df* = 1, 177, *p* < 0.0001 for plant and animal species, respectively). Thus, in highly invaded webs, many species interact with generalist aliens, and more species become highly dependent on them.

### Invasion and Web Connectivity

**Figure 4:**
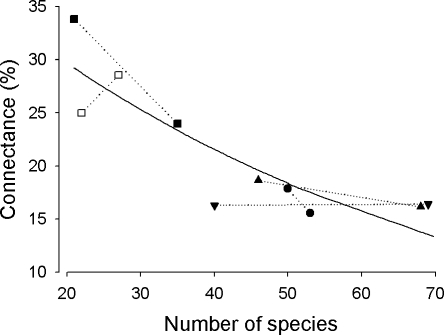
Relation of Connectance to the Number of Species Connectance represents the percentage of possible interactions that were actually recorded. The solid line indicates the best-fit negative exponential regression line (*y* = 40.9 × *e* ^–0.016x^, *F* = 221.6, *df* = 2, 8, *p* < 0.0001). *N* = 10 pollination webs. Symbols as in Figure 1.

**Figure 5:**
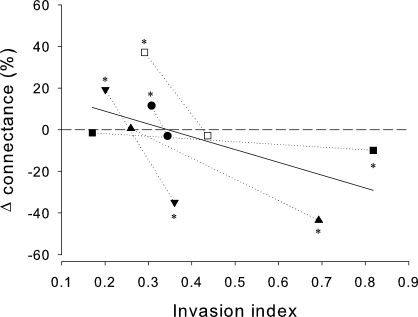
Relation of the Percent Difference in Connectance to Invasion Index Percent difference represents the connectance of the native plant-pollinator sub-web (*C_N_*) relative to the entire web (*C_C_*) and was estimated as 100 · 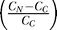 . Negative and positive connectance differences indicate that native sub-webs are less or more connected than their respective entire webs. We compared the connectances of the native sub-web and the sub-web formed by all other interactions by means of *χ* ^2^ test with *df* = 1. Asterisks indicate significant (*p* < 0.05) deviations. The solid line represents the best linear fit (*y* = 21.2 – 61.6*x*, *r* ^2^= 0.300, *F* = 3.41, *df* = 1, 8, *p* = 0.10). *N* = 10 pollination webs. Symbols as in Figure 1.

Changes in quantitative parameters characterizing the structure of invaded pollination webs were not associated with changes in network connectivity, but they were associated with an altered distribution of interaction links. Overall, the connectance (i.e., the percent of all possible interactions actually observed) of the analyzed webs decreased with the number of species in each web (Figure 4), a relation that could be depicted by a similar negative-exponential fit as the one estimated by Jordano from a sample of several qualitative plant–pollinator webs [24]. However, the remaining variation did not depend on the extent of web invasion (i.e., the invasion index; *r* ^2^ = 0.076, *F* = 0.66, *df* = 1, 8, *p* = 0.44) and paired comparisons of residuals between lightly and highly invaded webs were not significantly different (−1.13 ± 1.69 versus 1.23 ± 0.87 %; *t* = −1.05, *df* = 4, *p* = 0.35). In general, both groups of webs exhibited similar connectivity (22.1 ± 2.62 versus 20.1 ± 3.23 %, respectively; *t* = 0.99, *df* = 4, *p* = 0.38). However, native mutualists were more connected among themselves in the lightly invaded than in the highly invaded webs (25.2 ± 3.49 versus 16.8 ± 3.54%; *t* = 6.91, *df* = 4, *p* = 0.002), a difference that persisted (*p* = 0.04) after including the total number of native species as a covariate. The connectance of the native sub-webs exceeded or equaled the overall connectance exhibited by their respective webs in the lightly invaded networks, whereas it was significantly lower in three of the five highly invaded webs (Figure 5). For the other two highly invaded networks, where connectance among native species was similar to that exhibited by their respective webs, their lightly invaded counterparts were characterized by particularly richly connected native sub-webs. Thus, these results reveal general invariance in overall network connectivity, irrespective of invasion degree and beyond any influence of network size, so that if native species become less connected among themselves they become more connected with alien species.

## Discussion

Previous studies showed that alien mutualists can integrate into pollination webs, but with a slight effect, if any, on the connectivity of the original network [18,29]. Our results reveal, however, that the effect of aliens on the distribution of interaction links could greatly depend on the extent of invasion which, in turn, may be mediated by external factors, such as disturbance, and influenced by particular historical settings [9]. Our work also demonstrates that aliens can modify basic quantitative parameters of pollination webs, such as the strength of interactions and distribution of asymmetries, that constitute the basic foundations of the architecture of mutualistic networks [1,2,4–6].

Several recent studies propose that species persistence should increase in a mutualistic network governed by limited interdependency, as the disruption of any link will have little effect on community stability [1,5–7,30]. However, this low mutualism strength may characterize either a diffuse network dominated by weak mutual dependence between interacting pairs of species or a highly structured network dominated by asymmetric links with interacting partners depending unevenly on each other. Asymmetry seems common in plant–animal mutualistic networks [1] and was evident from the networks that we studied (Figure 2). This pattern reflects a type of mutualistic web in which specialists interact differentially with generalists, whereas weak mutual interactions between generalist partners characterize the left tail of the distribution. Our results also demonstrate that diminished mutualism strength results from the involvement of generalist alien species in an unusual proportion of the most asymmetric interactions in highly invaded webs. The strength achieved by some of these generalist aliens surpasses that of any native species, indicating that many species that are ecologically specialized because of being rare [23] interact exclusively with at least some of the invaders (see also [1,2]). Therefore, these super-generalist aliens become central nodes of highly invaded webs that might increase nestedness and the persistence of many species [4], but greatly modify network architecture during invasion.

Our results also suggest the likely dynamics of change in a pollination network during invasion. Upon arrival, alien newcomers are rare and probably persist in existing pollination networks through interactions with native generalists [17,18], so they engage in few interactions and exhibit limited integration due to their scarcity. If disturbance and/or the lack of regulatory processes, such as predation and parasitism, subsequently allow an increase in abundance of an alien species, its ecological generalization may also increase, because abundance is an important determinant of a species' degree [22,23,31]. Additionally, habitat modification can change the mode of action (i.e., per capita effect) of at least some alien species and thus their total impact may increase beyond expectations based on invader abundance alone [10]. Therefore, the growth of a population of an invasive species likely precipitates a disproportionate increase in its importance in the structure of the network through both numerical and functional effects. In the case of invasive plants, for instance, this augmented strength may result not only from high density, but also from the tendency of these species to produce exuberant flower displays and offer superabundant flower resources [12]. In the case of invasive flower visitors, this high species' strength may also relate to highly plastic behavior, efficient resource search and exploitation, and indiscriminate foraging as exhibited by the honey bee, Apis mellifera [32], and some highly invasive bumble bees such as Bombus terrestris [33] or B. ruderatus [34]. The conceptual model we portray may also accommodate contrasting evidence for the existence of the so-called “invader complexes”, groups of introduced species interacting more with each other than expected by chance [16,18,29,35]. Because interactions among super-generalist aliens form the main core of highly-invaded webs, facilitation between alien partners may become apparent only during late stages of invasion.

Whereas alien mutualists can integrate into native pollination networks, they seem not to increase the connectivity of the invaded web. Our results demonstrate that connectance decreased with total number of species in the same fashion as found in a previous and broader study [24], regardless of whether the networks are highly invaded (Figure 4). Indeed, the negative-exponential coefficient of the relation in Figure 4, 0.16, is practically the same as the 0.17 reported by Jordano [24]. This similarity can relate to different constraints that may determine limits in the connectivity of this type of network [30,36–38]. Therefore, the increasing role of aliens as prime nodes in network structure entails the erosion in connectivity among members of the pre-invaded web, as the decrease in connectivity among native species in some of the most invaded webs illustrates clearly (Figure 5). From the alien species' perspective, usurpation of links and of interaction frequency in mutualistic networks can establish a self-perpetuating positive feedback, whereby invasive species increasingly enhance their reproductive success and dominance. From the native species' perspective, whereas many of the “lost” interactions may be rather redundant, others might be key in their ecological and evolutionary dynamics. For instance, the clonal herb Alstroemeria aurea (Alstroemeriaceae) is a keystone mutualist in south Andean forests. Despite being highly generalist, its reproduction relies principally on the giant bumble bee B. dahlbomii, another key mutualist [39]. Recently, the introduced European bumble bee B. ruderatus invaded austral South America and displaced native B. dahlbomii even from some undisturbed areas [34,40]. Although the reproductive consequence of this pollinator replacement for *Al. aurea* is unknown, B. ruderatus is a less-efficient pollinator of this plant species because of its smaller size, and therefore pollination quantity and/or quality may decline [41]. In the long term, this novel interaction may select for smaller flowers of *Al. aurea*. For other native plant species, like the south Andean endemic Chilean fire-bush*, Embothrium coccineum* (Proteaceae), usurpation of links could significantly decrease reproductive output because of replacement of a diverse pollinator assemblage by *Ap. mellifera* in highly-modified settings [40]. In such cases, invasive species can contribute to their eventual demise through the disruption of the pollination mutualism (see also [15]).

Invaded mutualistic webs may actually sustain high diversity and even some rare pollinators may increase in abundance due to plentiful resources offered by some mass-flowering alien plants [42–44]. Yet, the focus on diversity of species and links or other metrics describing the structure of quantitative webs can overlook subtle, but transcendent changes in network architecture [45]. Although many native mutualists can survive alien dominance, our results indicate that particular configurations of biological communities, and thus unique ecological interactions and evolutionary pathways, can be lost forever.

## Materials and Methods

### The database.

We assembled a dataset (Dataset S1) composed of ten quantitative plant–pollinator webs, including alien species, from our unpublished records and Olesen et al. [29]. The field sampling procedures that we used to characterize the eight south Andean plant–pollinator webs are presented in detail elsewhere [16,35]. Briefly, per-flower visit frequencies (number of visits × flower^−1^ × 15 min^−1^) by different visitor species were estimated at the flowers of the most common animal-pollinated native and alien plant species at each of four forested sites in Nahuel Huapi National Park, Argentina, during the 2000–2001 flowering season. Sites were located along a 50-km transect, oriented East-West along a gradient of increasing precipitation. At each site, corresponding to a different native forest type, we surveyed flower-visiting animals in two habitats units <1 km apart characterized by contrasting disturbance intensity, a mostly undisturbed or less disturbed area (U) and a highly-disturbed area (D), which had been either burned or logged and was characterized by a higher number and abundance of alien species. In total, we accumulated 1,639 diurnal observation periods of 15 min each during 73 d. Sampling was distributed evenly among the four study sites, between disturbed and undisturbed habitats within sites and throughout the flowering season. Individuals observed visiting flowers were morphotyped and identified to the minimum possible taxonomic level with the aid of a reference collection and the expertise of different specialists. Individuals that could be identified to the species level accounted for about 85% of all flower visits recorded. Previous analyses conducted on this data set showed that plant species origin, alien versus native, influences visitation frequency and the composition of the flower-visiting species assemblage independent of habitat disturbance [16,35]. The data obtained from Olesen et al.'s study [29] of two other pollination webs from oceanic islands, Flores, in the Azores, and Ile aux Aigrettes, a small islet 600 m off the coast of Mauritius (hereafter Aigrettes), also included alien plants and pollinators. This study also involved a large sampling effort, including observations of the numbers of flower visitors seen during 226 and 341 observation periods of 30 min each on Flores and Aigrettes, respectively. However, Olesen et al. [29] did not contrast disturbed and undisturbed habitats on these islands. All flower visitors included in the ten sampled networks were presumed to act as pollinators, as they contacted sexual parts of the flowers they visited.

The plant species included in the networks encompass a mix of herbaceous and woody forms, whereas flower visitors were all insects, except for a hummingbird, Sephanoides sephaniodes, native to austral South America and present in some of the south Andean webs, and a gecko, *Phelsuma ornata ornata*, endemic to Mauritius. Eight out of the ten webs included the introduced honey bee, *Ap. mellifera*, whereas the bumble bee, B. ruderatus, was an alien flower visitor in six of eight south Andean webs [16] but native on Flores [29]. Although the south Andean webs share some native and alien species, each web includes unique species and species combinations (Dataset S1).

### Network parameters.

Each of the ten plant–pollinator webs was depicted in a matrix, in which the rows represent different plant species (P) and columns depict animal pollinators (A), and cells record the occurrence and intensity of interactions between plant and pollinator species. As measures of interaction frequency, we used the mean visit frequency per observation period for the south Andean matrices, and the total numbers of visits from different pollinator species during the total sampling period on each plant species for the island webs (see [2,23]). We calculated the connectance of each matrix (i.e., a measure of connectivity; [24]) as the percentage of the P × A cells with an interaction frequency >0.

Based on estimates of interaction frequency, we calculated the mutual dependency for all pairs of interacting species in each web. The frequency of an interaction relative to its row total (i.e., the fraction of all animal visits to a plant species by a particular animal species) represents the dependence 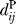 of plant species *i* on pollinator species *j*, whereas the frequency of a given interaction relative to its column total (i.e., the fraction of all visits by an animal species to a particular plant species) represents the dependence 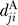 of pollinator species *j* on plant species *i*. We defined the strength of the mutualistic interaction between plant species *i* and pollinator species *j* as the product of their respective dependences 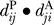 [1] and the mutualism strength for an entire web as the mean of all pairwise nonzero mutualistic strengths. A web dominated by either mutually weak or highly asymmetric interactions will exhibit low mutualism strength [1,28]. The observed mutualism strength was compared with a distribution of expected values generated from 10,000 randomized datasets, where we shuffled the observed interaction frequencies within each matrix with the restriction that each species had at least one interaction [2,23] (Protocol S1). Because of the sensitivity of mutualism strength to the number of species and links, we standardized the observed mutualism strength (*O*) for each web as (*O* – *E* 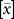 )/ *E* 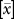 , where *E* 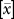 is the expected mean mutualism strength of its corresponding simulated distribution. This relative measure of mutualism strength was not influenced significantly by network size (Table 2). For each web, we estimated both the mutualism strength of the whole network and that of the native sub-web (i.e., after excluding interactions with and between alien species).

To identify the causes of changes in mutualism strength with increasing invasion, we compared the mean and distribution of the asymmetry between interacting pairs of native plant–pollinator species versus interacting pairs that included at least one alien species. Asymmetry between species *i* and *j* was characterized by AS(*i*, *j*) = 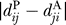 max 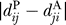 , which ranges between 0 and 1 [1].

## Supporting Information

Dataset S1 The Ten Pollination Networks (159 KB XLS)

Protocol S1 Matlab Code for Generating Randomized Distributions of Mutualism Strength (2 KB RTF)

## Abbreviations

*df*: degrees of freedom
